# Neutrophil gelatinase-associated lipocalin as a biomarker for predicting acute kidney injury after coronary artery bypass grafting

**DOI:** 10.1186/s42077-020-00123-5

**Published:** 2021-01-20

**Authors:** Haitham Mohy El Din Mahmoud Othman, Alaa Eid Mohamed Hassan, Mayar Hassan Elsersi, Ahmed Kamal Mohamed Ali Soliman, Dalia Fahmy Emam

**Affiliations:** Department of Anesthesiology, Intensive Care and Pain Management, Faculty of Medicine, Ain-Shams University, Cairo, 11591 Egypt

**Keywords:** Acute kidney injury, Serum creatinine, Neutrophil gelatinase-associated lipocalin, Cardiac surgery

## Abstract

**Background:**

Early and precocious determination of acute kidney injury (AKI) is essential to prevent morbidity and mortality following coronary artery bypass grafting (CABG). Evaluation of the perioperative renal function is substantial using novel biomarkers other than the late traditional method of using serum creatinine. Plasma neutrophil gelatinase-associated lipocalin (NGAL) is a biomarker investigated for early detection of AKI in patients undergoing coronary artery bypass grafting, and its role has to be determined in this study.

**Results:**

Twenty-five patients undergoing elective CABG were enrolled in this cohort study and were assigned into two groups: group I include the patients that did not develop AKI (no AKI group) and group II include the patients that developed AKI (AKI group). Acute kidney injury based on Kidney Disease: Improving Global Outcomes (KDIGO) classification had been developed in 7 patients (28%). Plasma NGAL levels at 6 h were higher in patients who developed AKI compared with those who did not (302 ± 88.02 vs. 116.50 ± 17.33 ng/m, *p* value < 0.001). The cut-off value of plasma NGAL levels measured 6 h postoperatively was 145 ng/ml and the area under the receiver-operating characteristic (ROC) curve was 0.965. Results of this study showed that plasma NGAL is a robust early biomarker of AKI, which preceded the rise in serum creatinine by many hours.

**Conclusion:**

This study revealed that earlier diagnosis of acute kidney injury in patients undergoing CABG can be achieved by measuring postoperative plasma NGAL concentration at 6 h.

## Background

Acute kidney injury (AKI) is a frequent intricacy after cardiac surgery. Cardiac surgery-associated kidney injury may affect 30% of the patients based on the literature and different definitions of acute kidney injury. Mortality increases from 1% up to half the patients who undergo hemodialysis following cardiac surgery (Thiele et al. 2015). AKI that follows CABG affects long-term survival negatively, even in patients with minor kidney dysfunction (Bouma et al. 2018).

Early identification of renal dysfunction is considered the most effective method to decrease the morbidity of postoperative AKI by permitting early diagnostic and therapeutic interventions to forestall renal failure development (Bataille et al. 2017).

Several hazard factors have been recognized to augment the incidence of AKI associated with cardiac surgery, including age, chronic hypertension, diabetes, and peripheral vascular disease are prevalent unmodifiable risk factors (Lopez-Delgado et al. 2013).

Other risk factors like utilization of cardiopulmonary bypass (CPB), transfusion of blood products (packed RBCs and fresh frozen plasma), and nephrotoxic drugs administration before or after surgery may as well increase the risk of postoperative AKI (Ostermann and Liu 2017).

Over the last decades, many endeavors have been made to seek out explicit biomarkers having the ability to anticipate acute damage of the epithelium of kidney tubules (Schrezenmeier et al. 2017).

Serum creatinine level has been accustomed to figure out changes in renal function for the past five decades. The half-life of serum creatinine in normal adult male is 4 h. After 50% decrease in creatinine clearance, the half-life of serum creatinine is around 8 h and it takes another three to five half-lives to reach peak level and reaching a steady state (it takes 24 to 40 h). This drawback causes serum creatinine to be a late marker for AKI even after decline in the glomerular filtration rate (GFR) to half (Srisawat and Kellum 2020).

Furthermore, the level of serum creatinine is affected by various factors, like gender, aging, the mass of the muscle, protein metabolism, medications, and fluid intake and deficit (Jain et al. 2016).

Because of the failure of the existing markers, more novel and reliable biomarkers that could work as ‘the kidney troponin’ should be considered, which may allow early identification of the damage of renal tubules, apart from the decrease in function (Abdallah et al. 2013).

Many novel biomarkers have been evaluated as possible biomarkers for early and meticulous AKI identification. These include biomarkers like NGAL that can be detected in plasma and urine, cystatin C which is detected in plasma, and urinary biomarkers like kidney injury molecule-1, urine NGAL, and interleukin-18 (de Geus et al. 2012).

NGAL is considered to be one of the most investigated AKI biomarkers. NGAL, otherwise known as Lipocalin-2, is a 25-kDa protein with 178 amino acids. Plasma NGAL is produced by neutrophils and filtered by the glomeruli freely and afterward completely reabsorbed by the proximal convoluted tubules (Chew and Hwang 2019). Expression of NGAL is induced by tissue injury and its expression is quite prompt, usually within 2–4 h of injury. Fortunately, NGAL is very stable and easily detected in serum and urine (Kashani et al. 2017).

NGAL was recently proposed as a portentous early predictive biomarker for kidney injury (Rysz et al. 2017).

## Methods

This prospective cohort study was conducted in Ain Shams University hospitals from January 2019 to December 2019.

After ethical committee approval, written informed consent had been taken from 25 participants of either sex with normal preoperative creatinine level (0.6–1.2 mg/dl), scheduled for elective coronary artery bypass grafting (CABG) using cardiopulmonary bypass (CPB).

Exclusion criteria included: age < 18 years, emergency surgery, Redo operations, pre-existing renal impairment, or renal replacement therapy (RRT), low ejection fraction < 30%, the need for postoperative intra-aortic balloon, chronic inflammatory disorders and immunosuppression, corticosteroid therapy, and patient refusal.

### Study interventions

Demographic data, patients’ risk factors, preoperative evaluation of all the patients enrolled in the study and preoperative routine laboratory test as coagulation profile, CBC, kidney function, and liver function tests were evaluated and recorded. All the patients continued the preoperative medications till the night of surgery with the exception of nephrotoxic drugs. Upon arrival to the operating room, peripheral venous cannula was inserted and radial artery cannulation was done in the non-dominant hand under local anesthesia after checking the patency of the collaterals through the modified Allen’s test. Two milligrams midazolam and 4 mg morphine IV were given for sedation after application of standard monitors to the patient according to the American Society of Anesthesiologist (ASA) recommendations. Induction of anesthesia was done using fentanyl at 5 μg/kg, midazolam at 0.05 mg/kg (titrated dose), thiopental at 3–5 mg/kg (titrated dose), and rocuronium at 0.9 mg/kg to achieve muscle relaxation in order to facilitate tracheal intubation. Blood pressure was maintained within 20% of the baseline with avoiding tachycardia (keeping HR between 60–80 bpm) during induction. Maintenance of anesthesia was done by isoflurane (1.2%), fentanyl and rocuronium.

Rt. Central venous line was inserted by the guidance of ultrasound.

Anticoagulation was done before cannulation of the aorta using intravenous heparin 300 to 400 units/kg with target ACT between 400 and 480 s.

After clamping of the aorta, antegrade cold cardioplegia (each 1 L ringer solution contained 30 mmol/l potassium chloride, 100 mg lidocaine, and 1 gm magnesium sulfate) was administered in a dose 10 mg/kg. Cardioplegia was given in a dose of 5 mg/kg every 20 minutes.

CPB was established to keep the mean arterial pressure (MAP) during full flow between 50 and 70 mmHg. Hematocrit was kept above 20%, fresh packed RBCs were given if needed. CBP time and aortic cross-clamping time were measured and recorded.

Patients were weaned from the CPB according to the general condition of the patient and cross-clamp time. After venous and arterial decannulation, protamine was administered for reversal of heparin (1 mg per 100 units of totally administered heparin, slowly to avoid hypotension). The patients enrolled in the study were weaned from the ventilator in the intensive care unit (ICU) after vital data stabilization (MAP above 60 mmHg to 90 mmHg, heart rate (HR) within normal range above 60 and below 90 bpm and central venous pressure (CVP) within the range of 10 and 15 cm H_2_O) and fulfillment of the criteria for extubation. Patients were followed up and managed for the 48 h study period as per standard protocols.

NGAL levels were determined at two time points: preoperatively (within 24 h before the surgery) and postoperatively 6 h after arrival to the ICU. Assay of serum NGAL was done by the enzyme-linked immune sorbent assay (ELISA) method, using the kit supplied by Shanghai Sunred Biological Technology Co., Ltd. The kit uses a double-antibody sandwich enzyme-linked immunosorbent assay (ELISA).

Serum creatinine levels were measured at five time points: preoperatively before induction of anesthesia and at 6, 12, 24, and 48 h postoperative. Serum creatinine concentration was measured using a Beckman® Analyzer (Beckman Coulter, Brea, CA, USA) according to the picric acid colorimetric method.

Postoperative AKI during the 48 h follow-up period was determined based on the Kidney Disease: Improving Global Outcomes (KDIGO) classification and the levels of the 6 h postoperative plasma NGAL values for renal impairment were checked. Other variables we included were age, gender, aortic cross clamp time, and CPB time.

### Stages of AKI based on KDIGO classification (Khwaja 2012)

- Stage 1 AKI was defined as rise in serum creatinine by 0.3 mg/dL or by an increase ≥ 1.5-fold from the baseline value
- Stage 2 AKI classified by a 2–2.9-fold rise in baseline serum creatinine
- Stage 3 AKI by ≥ 3-fold rise in serum creatinine.

The primary outcome of this study was to evaluate the efficiency of neutrophil gelatinase-associated lipocalin (NGAL) as a biomarker in predicting AKI in patients scheduled for elective coronary artery bypass grafting (CABG) under cardiopulmonary bypass (CPB).

### Sample size

Using MedCalc program version 11 setting alpha error at 5% and power 90% results from previous study, (Prabhu et al. 2010) showed a significant correlation between neutrophil gelatinase-associated lipocalin (NGAL) and change in serum creatinine (*r* = 0.8). Based on this and taking in consideration 15% drop out rate, the needed sample is 25 patients.

This study was done as a prospective cohort study, in which 25 patients included.

The patients were assigned into two groups:

- Group I include the patients that did not develop AKI (no AKI group)
- Group II include the patients that developed AKI (AKI group)

### Statistical package and analysis

The data was collected, revised, coded, and introduced to a PC using statistical package for social science (SPSS 20.0. for windows; Armonk, NY: IBM Corp., 2010). Data was presented as mean and standard deviation (±SD) for quantitative prometric data. Independent *t* test was used to compare quantitative variables between the two study groups when the data were normally distributed. Qualitative data were presented as number and percentage and the differences between the two groups were compared using the chi-square (*χ2*) test and/or Fisher exact test when the expected count in any cell found less than 5. *p* < 0.05 will be considered significant.

Pearson correlation coefficient was used to test correlations between serum NGAL and other variables.

The receiver operating characteristic (ROC) curve and area under the curve (AUC; with 95% confidence intervals [CIs]) of plasma NGAL measured 6 h postoperatively were calculated and compared for the assessment of its ability to anticipate acute kidney injury.

## Results

Twenty-five patients without pre-existing renal dysfunction undergoing elective on pump CABG who did not have exclusion criteria were enrolled in this prospective cohort study.

The kidney injury was determined based on kidney disease improving global outcomes (KDIGO) classification in terms of rise in serum creatinine during the first 2 days after surgery.

Of the 25 patients studied, 7 (28%) had AKI postoperatively as defined by KDIGO criteria while 18 (72%) did not, by measuring serum creatinine (SCr) 48 h postoperatively.

Six patients who had AKI developed stage 1 AKI while only one patient had stage 2 AKI. None of the patients of AKI group developed stage 3 AKI or underwent dialysis.

Average age was 56.54 ± 6.44 years in No AKI Group vs. 60.67 ± 4.51 years in AKI Group with (*p* value 0.136)

There is statistically non-significant difference between AKI group and No AKI group as regards age and sex where *p* value was > 0.05 (*p* value 0.284).

Patients’ characteristics regarding body mass index, medical history of diabetes mellitus, familial history of coronary artery disease (CAD), history of dyslipidemia, diuretics use before surgery, preoperative hemoglobin, and preoperative left ventricular systolic function were comparable in both groups (Table 1)

**Table 1:** Basic demographics and clinical data of both groups

Patient characteristics	No AKI	AKI	***p*** value
Age	56.54 ± 6.44	60.67 ± 4.51	0.136
Male sex (%)	16 (89%)	5 (71%)	0.284
BMI (kg/m^2^)	25.44 ± 2.04	26.29 ± 1.80	0.349
Diabetes (%)	8 (44%)	4 (57%)	0.568
Dyslipidemia (%)	11 (61%)	4 (57%)	0.855
History of hypertension (%)	6 (33%)	6 (85%)	0.019*
Family history of CAD (%)	3 (17%)	2 (29%)	0.504
LVEF	60.33 ± 3.02	58.85 ± 2.73	0.272
Diuretics use before surgery (%)	6 (33%)	2 (29%)	0.818
COPD (%)	2 (11%)	1 (14%)	0.826
Smoker (%)	12 (67%)	4 (57%)	0.656
Preoperative hemoglobin (gm/dl)	13.71 ± 1.15	13.27 ± 0.71	0.365
ICU stay (days)	1.56 ± 0.61	2.86 ± 0.69	< 0.001*
Preoperative plasma creatinine (mg/dl)	0.89 ± 0.07	0.91 ± 0.07	0.65
Preoperative plasma NGAL (ng/ml)	78.44 ± 7.35	78.71 ± 4.75	0.914

Data presented as mean ± SD

*p* value < 0.05 was considered statistically non-significant

*p value < 0.001 HS

The number of hypertensive patients in the AKI group (85%) compared to the no AKI group (33%) was significantly higher (*p* value = 0.019)

AKI group had a longer ICU stay compared to the no AKI group

There is no statistical difference as regards preoperative serum creatinine and serum NGAL levels between the two groups where *p* value was > 0.05 (Table 1)

Patients with AKI had longer CPB time (109.42 ± 7.48 vs. 84.00 ± 6.87 min) (*p* value < 0.001) and longer aortic cross-clamp time (93.86 ± 6.77 vs. 69.4 ± 6.49 min) (*p* value < 0.001) compared to the other group (Table 2).

**Table 2:** Comparison of aortic and cross clamping time in both groups

Patient characteristics	No AKI	AKI	***p*** value
Bypass time (min)	84 ± 6.87	109.42 ± 7.48	< 0.001*
Aortic cross clamping time (min)	69.40 ± 6.49	93.86 ± 6.77	< 0.001*

Data presented as mean ± SD

*p* value > 0.05 was considered statistically non-significant

*p value < 0.001 is highly significant

**Fig. 1:**
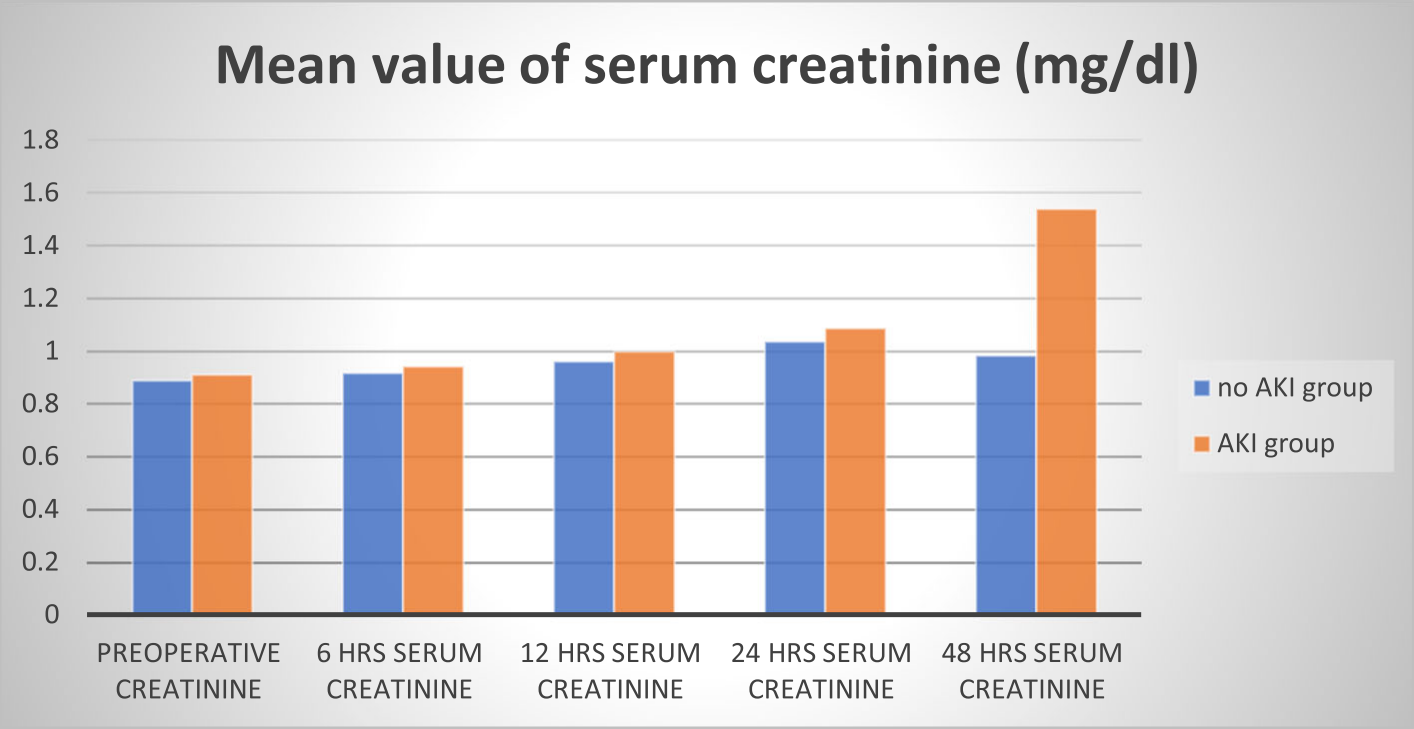
Mean value of serum creatinine in both groups

No significant difference between SCr (*p* value > 0.05) in the two groups for preoperative levels and postoperative creatinine levels at 6, 12, and 24 h. The AKI group had higher postoperative 48 h SCr levels (*p* < 0.001) as shown in (Fig. 1).

**Fig. 2:**
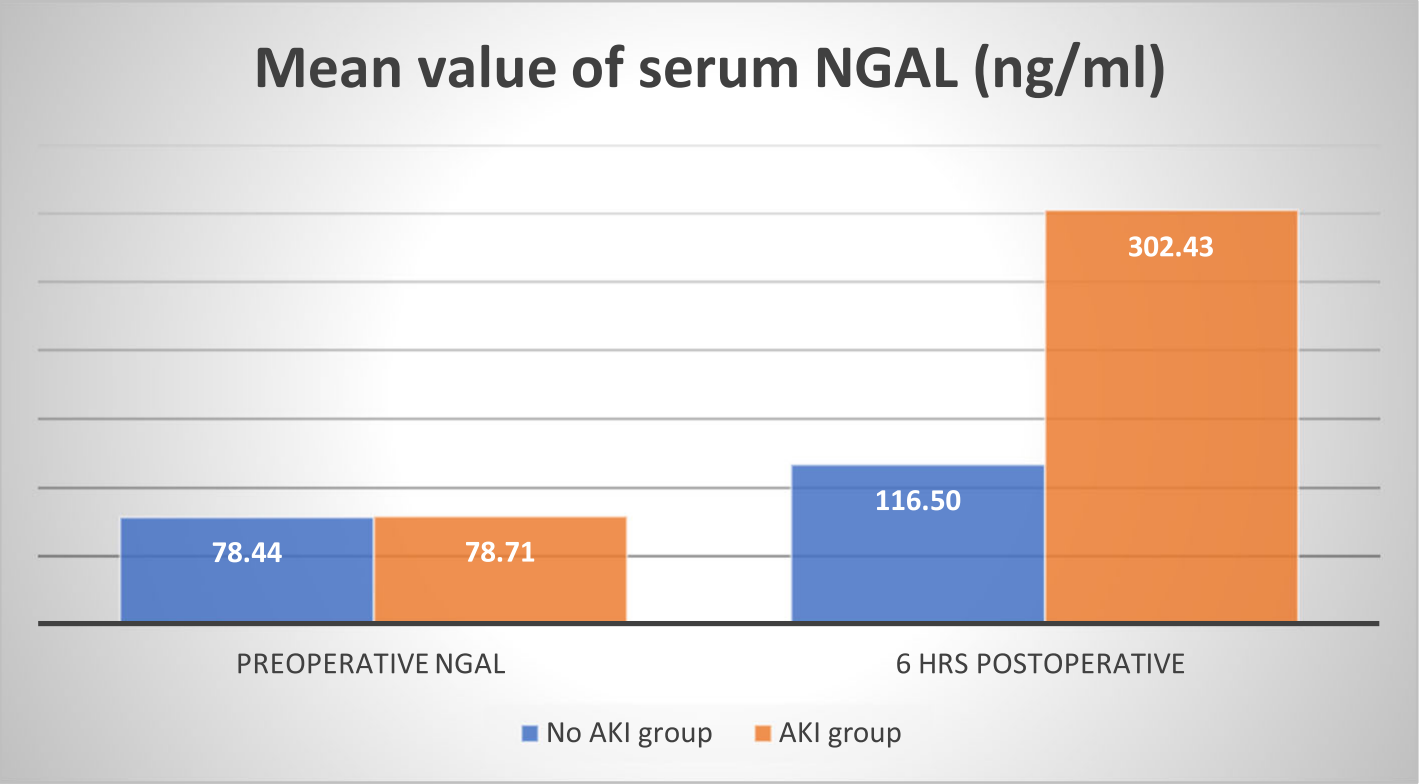
Mean value of plasma NGAL in both groups

Measurement of plasma NGAL 6 h postoperatively showed higher levels in patients who had AKI during the first 2 days follow-up vs those who did not (302 ± 88.02 vs. 116.50 ± 17.33 ng/mL) (*p* value < 0.001) as shown in Fig. 2.

In the AKI group, there is strong positive correlation between 6 h serum NGAL levels and serum creatinine levels measured 48 h after surgery(*r* = 0.857) (*p* value = 0.014) (Table 3)

**Table 3:** Correlation between serum NGAL 6 h postoperative and change in creatinine in the AKI group after 2 days in the AKI group

Parameter	Mean ± SD	Correlation coefficient (*r*)**	*p* value
Creatinine level at 6 h	0.94 ± 0.05	− 0.109	0.82
Creatinine level at 12 h	1 ± 0.06	− 0.045	0.92
Creatinine level at 24 h	1.09 ± 0.07	0.33	0.47
Creatinine level at 48 h	1.53 ± 0.09	0.857	0.014*

*p value less than 0.05 is considered statistically significant *p* value < 0.001 is highly significant

**Pearson’s rank correlation coefficient

**Fig. 3:**
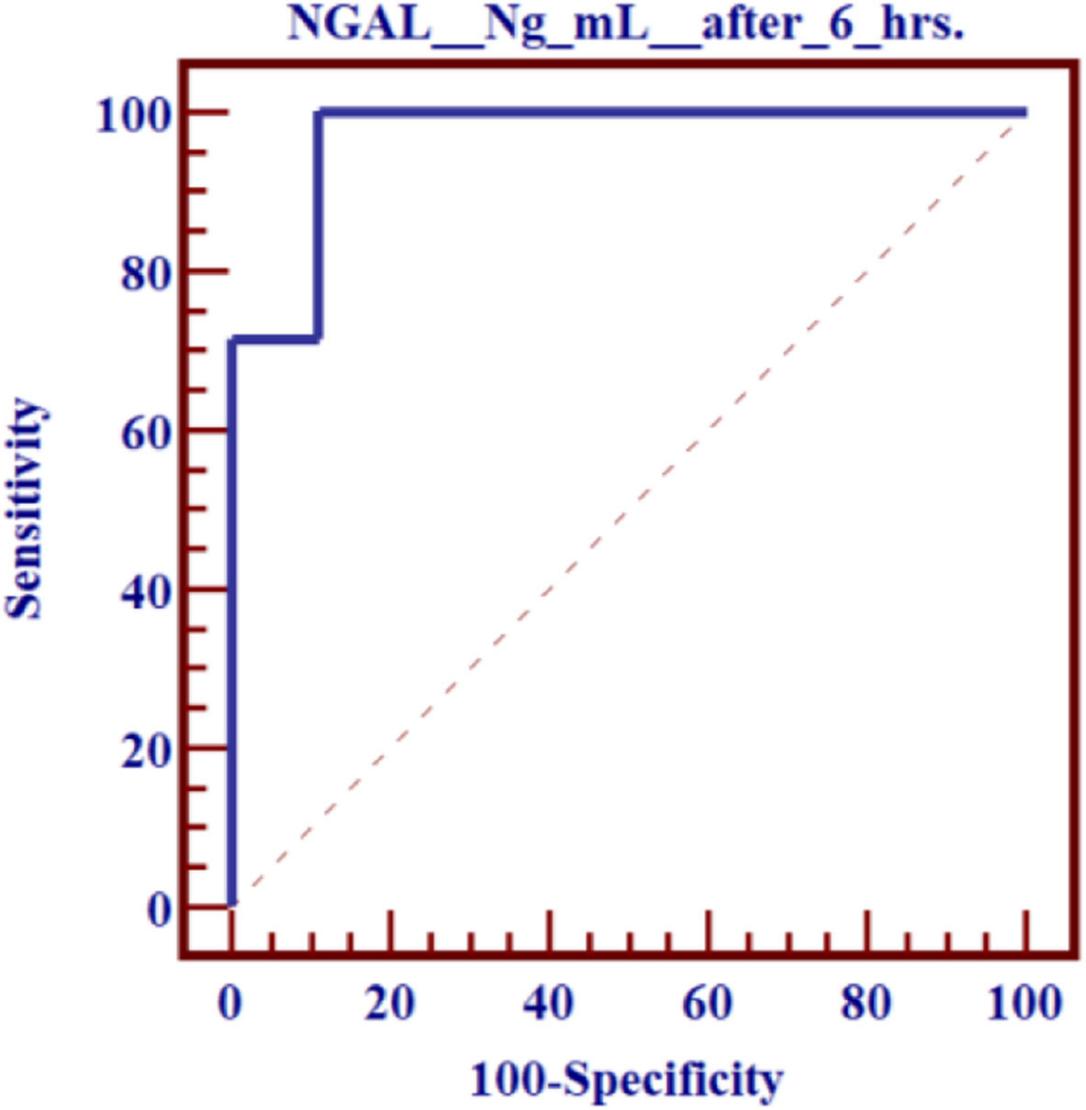
Receiver-operating characteristic (ROC) curve of plasma NGAL level (cut-off value = 145 ng/mL) at 6 h after operation. AUC = area under the curve

The cut-off value of the 6 h postoperative plasma neutrophil gelatinase-associated lipocalin levels was 145 ng/ml and the area under the receiver-operating characteristic curve was 0.965 as shown in Fig. 3. The sensitivity, specificity, and negative and positive predictive values of plasma NGAL after 6 h of operation in early detection of kidney injury in the postoperative 2 days period were found to be 100%, 88.9%, 100%, and 77.8%, respectively (Table 4)

**Table 4:** Sensitivity and specificity of plasma NGAL (at cut-off value > 145 ng/ml)

ROC curve between AKI and No AKI as regards NGAL (ng/ml)					
Cut-off	Sens.	Spec.	PPV	NPV	Accuracy
> 145 *	100.0	88.9	77.8	100.0	96.8

## Discussion

Acute kidney injury following cardiac surgery is a frequent and serious complication, it is considered to be independently associated with worse outcomes, increased mortality rate, prolonged ICU, and hospital stay (Harky et al. 2020).

Serum creatinine increases only when more than 50% of renal function is lost; in addition, several factors influence creatinine levels making it an inadequate marker for AKI (Srisawat and Kellum 2020). Therefore, there is a crucial need for more accurate and reliable biomarkers that might result in early prediction of AKI and therefore help timely initiation of appropriate therapeutic interventions or applying preventive measures.

In several preclinical and clinical studies, NGAL showed promising performance in detecting AKI hours earlier than serum creatinine and was postulated to be the “troponin” of the kidneys (Abdallah et al. 2013).

This prospective cohort study was done in Ain Shams University Hospitals for a period of 1 year. Twenty-five patients scheduled for elective CABG were enrolled in this study.

Four (16%) women and 21 (84%) men patients were enrolled in the study. The mean age was 57.69 ± 6.17 years.

In all patients enrolled in our study, serum NGAL was measured at two time points: preoperative within 24 h before surgery and 6 h postoperatively in all patients enrolled in this study

Serum creatinine was measured preoperatively at 6 h, 12 h, 24 h, and 48 h after surgery.

A total of 7 patients out of 25 (28%) developed AKI according to Kidney Disease: Improving Global Outcomes (KDIGO) KDIGO criteria, plasma NGAL levels at 6 h postoperative were significantly higher in all patients with AKI compared to their baseline levels, whereas only one patient had elevated serum creatinine at 6 h postoperative.

Our data suggests that plasma NGAL is a robust early AKI biomarker, which forgoes the rise in serum creatinine by many hours allowing the avoidance of nephrotoxic drugs (antibiotics and nonsteroidal anti-inflammatory drugs) and dose adjustment of the antibiotics; therefore, decreasing the morbidity and the necessity of RRT.

Plasma NGAL measured 6 h postoperatively showed higher levels in patients who had AKI during the first 2 days follow-up vs those who did not (302 ± 88.02 vs. 116.50 ± 17.33 ng/mL) (*p* value < 0.001). In AKI group, there was a statistically significant correlation between 6 h NGAL level and creatinine measured 48 h after surgery (*r* = 0.859) (*p* value = 0.014)

The ROC analysis of this study revealed that the cut-off values for NGAL measured 6 h after procedure was 145 ng/mL and the AUC for the ROC curve was 0.965, with sensitivity (100%) and specificity (88.9%).

Our results are in line with several previous studies that suggested NGAL as a highly promising biomarker for predicting AKI early after cardiac operations.

Mishra et al. measured NGAL levels in the pediatric population undergoing cardiac surgery and reported that serum NGAL showed a 10-fold increase 2 h postoperative in patients who developed AKI later (Mishra et al. 2005). Further studies were performed in children undergoing cardiac surgical procedures: Dent et al. showed comparable finding by demonstrating a significant rise of plasma NGAL at 3 h after CPB (Dent et al. 2007). Fadel et al. also found that plasma NGAL measurements may be efficient for early detection of kidney damage triggered by CPB (Fadel et al. 2012).

Our findings were also similar to that of the study carried out by Miah et al., that demonstrated that plasma NGAL level 6 h after completion of cardiac valve surgery increased prior to serum creatinine level rise and thus AKI can be detected earlier by plasma NGAL (Miah et al. 2018).

In agreement with our study, Tuladhar et al. found that there was a marked rise in plasma NGAL level in all participants and this increase in the levels after cardiac surgery was significantly higher in patients with AKI in comparison to those without AKI (Tuladhar et al. 2009).

Similar results were observed in the prospective cohort study done by Haase et al. on adult patients at risk of AKI due to cardiac surgery and other causes, they found that plasma NGAL 6 h after CPB fulfilled several criteria of a promising renal biomarker and the increase in plasma NGAL preceded the rise in serum creatinine or the clinical diagnosis of AKI (Haase et al. 2011).

Recent studies have provided important findings about cut-off values consistent with this study; Ghonemy and Amro found that the sensitivity and specificity of serum NGAL at 3 h after the end of surgery was 94.1% and 93.9% respectively. After 6 h post-operative, NGAL sensitivity increased to 98.1% with minor decline in the specificity to 91.9% (Ghonemy and Amro 2014).

Similarly, Park et al. found that plasma NGAL levels were significantly increased early at immediate postoperative ICU admission, while serum creatinine showed no significant increase. They determined serum NGAL cut-off value of 165.5 ng/ml on ICU admission with 61.5% sensitivity and 88.9% specificity (Park et al. 2015).

Similar results were reported by Parikh et al., and they revealed that peak plasma NGAL levels were throughout the first 6 h following surgery. Elevated plasma NGAL levels significantly predicted which patients were at higher risk of developing AKI, with area under the receiver-operating characteristic (AUC) curve of 0.75 (Parikh et al. 2011).

Another study conducted on patients with chronic kidney impairment scheduled for cardiac surgery; Perrotti and his colleagues found that serum NGAL levels greater than 155 ng/ml, measured 6 h after CPB were considered to be independent predictor of AKI, with a sensitivity of 79% and specificity of 58%, suggesting that its predictive value is not affected by baseline renal function (Perrotti et al. 2015).

There was also another study in accordance with this result as in study proclaimed by Prabhu et al. that found that plasma NGAL measurement in patients 4 h following CPB predicted AKI at a cut-off value 229 ng/mL with an area under the curve of 0.98 (Prabhu et al. 2010).

In the meta-analysis done by Zhou et al. including 24 cohort studies (22 prospective and 2 retrospective studies), NGAL level measured within 6 h postoperative was an adequate biomarker for early detection of AKI (Zhou et al. 2016).

However, conflicting data to our results were reported by Friedrich et al. they included 81 patients undergoing CBP and conducted that although NGAL values increased postoperatively, the peak values failed to prognosticate the development of AKI or the severity of injury (Friedrich et al. 2017). Similarly, Perry et al. found that the increase in plasma NGAL levels was associated with AKI; however, its utility was limited due to its low sensitivity (38.7%) (Perry et al. 2010). Different explanations for these contradicting conclusions may be the difference in the timing of sampling, patients’ demographics and comorbidities, and the AKI definition used in these studies.

Our study emphasized that prolonged CPB time is associated with occurrence of AKI postoperatively, as mentioned by Boldt et al. who stated that patients with cardiopulmonary bypass time greater than 90 min showed greater kidney injury than patients with bypass time less than 70 min (Boldt et al. 2003).

Also Karim et al**.** found that the incidence of AKI increased along with the rise in the CPB and cross clamp time significantly. The CPB time more than seventy minutes increased the AKI hazard by an odds ratio (OR) 4.76 as compared to 71–140 min and by an OR 6.30 for > 140 min (*p* value < 0.01) while for the cross clamp time more than 60 min increased the AKI hazard by an OR of 2.84 as compared to 61–120 min and by an OR 3.64 for > 120 min (*p* = 0.01) (Karim et al. 2017).

## Limitations

The small group number (*n* = 25) included in the study is considered to be an important limitation. To evaluate the ability of plasma NGAL for earlier diagnosis of AKI after coronary artery bypass grafting, larger randomized prospective trials are required.

The other limitation of this study is that the plasma NGAL level was assessed at 6 h postoperatively and not after that, which is considered a short observational period. In spite of the fact that earlier studies on NGAL have showed that AKI and the elevation of plasma NGAL levels occur in the majority of studies during the aforementioned period.

Despite normal serum creatinine levels before surgery for all the patients enrolled in the study, measurement of the glomerular filtration rate before surgery to evaluate kidney function was not done.

## Conclusion

This study revealed that early prediction of acute kidney injury in patients undergoing CABG can be achieved by monitoring plasma NGAL levels at baseline and 6 h after surgery. The early rise in plasma NGAL levels, preceding serum creatinine can be beneficial in providing an early warning to critical care providers allowing the possibility of taking earlier interventions in this high risk population.

## Data Availability

The datasets used and/or analyzed during the current study are available from the corresponding author on reasonable request.

## Abbreviations

AKI: Acute kidney injury
ASA: American Society of Anesthesiologist
CABG: Coronary artery bypass grafting
CAD: Coronary artery disease
CPB: Cardiopulmonary bypass
CVP: Central venous pressure
GFR: Glomerular filtration rate
HR: Heart rate
KDIGO: Kidney Disease: Improving Global Outcomes
LVEF: Left ventricular ejection fraction
MAP: Mean arterial pressure
NGAL: Neutrophil gelatinase-associated lipocalin
ROC: Receiver operating characteristic
RRT: Renal replacement therapy
SCr: Serum creatinine
